# Antiepileptic Stiripentol May Influence Bones

**DOI:** 10.3390/ijms22137162

**Published:** 2021-07-02

**Authors:** Agnieszka Matuszewska, Beata Nowak, Anna Nikodem, Anna Merwid-Ląd, Benita Wiatrak, Tomasz Tomkalski, Diana Jędrzejuk, Ewa Szeląg, Tomasz Sozański, Maciej Danielewski, Paulina Jawień, Ireneusz Ceremuga, Marta Szandruk-Bender, Marek Bolanowski, Jarosław Filipiak, Adam Szeląg

**Affiliations:** 1 Department of Pharmacology, Wroclaw Medical University, ul. Jana Mikulicza-Radeckiego 2, 50-345 Wrocław, Poland; beata.nowak@umed.wroc.pl (B.N.); anna.merwid-lad@umed.wroc.pl (A.M.-L.); benita.wiatrak@umed.wroc.pl (B.W.); tomasz.sozanski@umed.wroc.pl (T.S.); maciej.danielewski@umed.wroc.pl (M.D.); paulina.jawien@umed.wroc.pl (P.J.); marta.szandruk-bender@umed.wroc.pl (M.S.-B.); adam.szelag@umed.wroc.pl (A.S.); 2 Department of Mechanics, Materials and Biomedical Engineering, Wroclaw University of Science and Technology, ul. Ignacego Łukasiewicza 7/9, 50-371 Wrocław, Poland; anna.nikodem@pwr.edu.pl (A.N.); jaroslaw.filipiak@pwr.edu.pl (J.F.); 3 Department of Endocrinology, Diabetology and Internal Medicine, Tadeusz Marciniak Lower Silesia Specialist Hospital—Centre for Medical Emergency, ul. Gen. Augusta Emila Fieldorfa 2, 54-049 Wrocław, Poland; tomkalski@onet.eu; 4 Department of Endocrinology, Diabetes and Isotope Therapy, Wroclaw Medical University, wyb. Ludwika Pasteura 4, 50-367 Wrocław, Poland; diana.jedrzejuk@umed.wroc.pl (D.J.); marek.bolanowski@umed.wroc.pl (M.B.); 5 Department of Maxillofacial Orthopedics and Orthodontics Institute, Wroclaw University of Science and Technology, ul. Krakowska 26, 50-425 Wrocław, Poland; ewa.szelag@umed.wroc.pl; 6 Department of Medical Biochemistry, Wroclaw Medical University, ul. Tytusa Chałubińskiego 10, 50-368 Wrocław, Poland; ireneusz.ceremuga@umed.wroc.pl

**Keywords:** antiepileptic drug, bone, computed tomography, stiripentol, vitamin D

## Abstract

Bone structure abnormalities are increasingly observed in patients chronically treated with antiepileptic drugs (AEDs). The majority of the available data concern older conventional AEDs, while the amount of information regarding newer AEDs, including stiripentol, is limited. The aim of the study was to assess the effect of stiripentol on bones. For 24 weeks, male Wistar rats, received 0.9% sodium chloride (control group) or stiripentol (200 mg/kg/day) (STP group). In the 16th week of the study, we detected lower serum PINP levels in the STP group compared to the control group. In the 24th week, a statistically significant lower 1,25-dihydroxyvitamin D_3_ level, higher inorganic phosphate level and higher neutrophil gelatinase-associated lipocalin (NGAL) levels in serum were found in the STP group compared to the control. Micro X-ray computed tomography of the tibias demonstrated lower bone volume fraction, lower trabecular thickness, higher trabecular pattern factor and a higher structure model index in the stiripentol group. Considering the results of this experiment on rats which suggests that long-term administration of stiripentol may impair the cancellous bone microarchitecture, further prospective human studies seem to be justified. However, monitoring plasma vitamin D, calcium, inorganic phosphate and kidney function in patients on long-term stiripentol therapy may be suggested.

## 1. Introduction

Epilepsy is one of the most common diseases of the central nervous system. The use of antiepileptic drugs (AEDs) is the basic treatment method [1]. Regular use of antiepileptic drugs is often necessary to control epileptic seizures. AEDs are usually taken chronically for many years and even for the rest of a patient’s life. However, seizures cannot be fully controlled in about one-third of patients with epilepsy [2]. Stiripentol is one of the drugs used in drug-resistant epilepsy [3].

Numerous studies confirm that epilepsy patients are at a higher risk of bone fractures [4,5]. The etiology of this phenomenon is multifactorial and involves the effect of the disease itself, an increase in the rate of falls, a lower bone strength and the adverse effects of pharmacotherapy, among others factors [4,6]. Approximately 50% of patients treated with antiepileptic drugs present bone structure abnormalities [1]. Rickets, osteomalacia and osteoporosis were observed in patients taking AEDs. In particular, older conventional AEDs inducing enzymes of cytochrome P450 are known to reduce bone mineral density [4]. AEDs inducing cytochrome P450 (e.g., phenytoin, carbamazepine and phenobarbital) may increase catabolism of vitamin D resulting in hypocalcaemia that induces the release of the parathyroid hormone, which promotes a decrease in bone mass [1]. Newer antiepileptic drugs seem to be better tolerated and to have a more favorable safety profile compared with conventional AEDs [3], but their effect on bone metabolism requires further investigation as the data available are contradictory [4,7,8].

Stiripentol (STP) has a unique chemical structure that is unrelated to other AEDs [9]. Available data suggest several possible mechanisms of the STP anticonvulsant effect [10]. STP inhibits the synaptic uptake of gamma-amino butyric acid (GABA), increases its release and extends the duration of activation of GABA_A_ receptors [11]. Stiripentol inhibit the activity of lactate dehydrogenase, which results in a decreased ATP level in neurons and, subsequently, neuronal hyperpolarization [10]. STP may also have neuroprotective effect [10,12]. The other mechanism of action involves a pharmacokinetic interaction with simultaneously used drugs, such as clobazam and valproate. STP intensifies the effects of these drugs by inhibiting the activity of cytochrome P450 isoenzymes that metabolize typical antiepileptic drugs [13,14].

Stiripentol, in combination with clobazam and valproic acid, is effective in the treatment of the Dravet syndrome and, in the majority of cases, reduces both seizure severity and frequency. The benefits of stiripentol administration in the management of malignant migrating partial seizures in infancy, intractable focal epilepsy or super-refractory status epilepticus were suggested after analyzing a small case series [10].

Dravet syndrome is a severe epileptic encephalopathy of childhood which is genetically determined [15]. After a period of normal development, children initially considered as healthy developed epileptic seizures (usually in the first year of life) [16]. The frequency of seizures increases in children in the first decade of their life. Later, the frequency of various seizures such as myoclonic, atypical absences or focal seizures with impaired awareness tends to decrease during adolescence and in adults [17]. In a few cases, Dravet syndrome can also be diagnosed in adulthood [18].

Childhood is a key period for obtaining the peak bone mass. The magnitude of peak bone mass determines the risk of osteoporosis and pathological fractures in adulthood [19]. In patients with Dravet syndrome, short stature and low levels of insulin-like growth factor 1 (IGF-1) were found [20]. The etiology of these findings is neither clear nor fully explained. The impact of the disease (epilepsy) and the potential influence of the treatment administered are considered. Stiripentol is used in children and adults with Dravet syndrome [17]. In the literature, there are no studies analyzing the effect of stiripentol on bones. In humans, STP is always used together with other drugs [3], which largely hinders the assessment of the drug’s individual effect on tissue. Therefore, we examined the effect of stiripentol alone on bones in rats. The rat model is an optimal animal experimental model for the evaluation of the impact of drugs on bone [21,22,23]. In order to eliminate the impact of epilepsy on the parameters analyzed, epilepsy-free rats were used in the study.

## 2. Results

### 2.1. Body Weight

**Figure 1:**
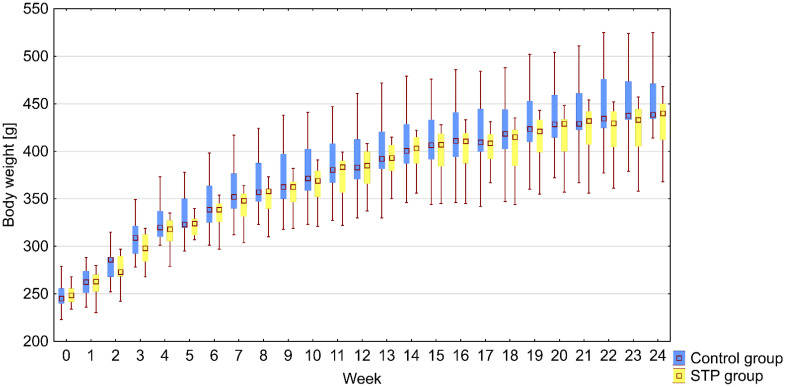
The effect of administration of stiripentol on body weight in rats. Results presented as follows: median (lower quartile—upper quartile).

Prior to stiripentol and/or normal saline being administered, both experimental groups were not significantly different in terms of body weight. The groups were compared in terms of body weight during the whole time of the experiment and on the last day of the experiment (Figure 1).

### 2.2. Serum Parameters

The parameters measured in the serum are shown in Table 1.

In the 16th week of the study, we detected a lower serum PINP level in the STP group compared to the control group.

In the 24th week of the study, a statistically significant lower level of 1,25-dihydroxyvitamin D_3_, a higher level of inorganic phosphate and a higher level of neutrophil gelatinase-associated lipocalin (NGAL) were observed in the group receiving stiripentol.

### 2.3. Bone Macrometric Parameters

At the end of the study, no statistically significant difference in weight and dimensions (length and diameter) of the right-side tibia and the right-side femur was observed between the groups. The results are summarized in Table 2.

### 2.4. Bone Mineral Density

After 24 weeks, no significant difference in terms of BMD was detected (Table 3).

### 2.5. Micro X-ray Computed Tomography

Statistically significant lower bone volume fraction, lower trabecular thickness, higher trabecular pattern factor and higher structure model index were observed in the tibias from the animals belonging to the group receiving stiripentol.

**Figure 2:**
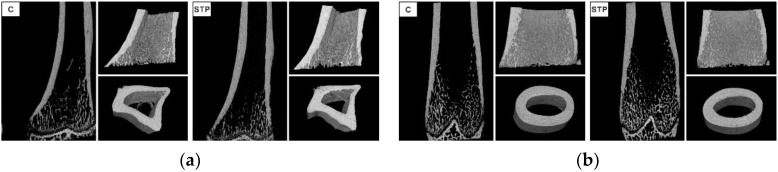
Sample micro X-ray computed tomography images of (**a**) tibia and (**b**) femur (C—control group; STP—group that received stiripentol for 24 weeks).

No differences in parameters determined for the femurs and the third lumbar vertebra were observed between the STP group and the control. The results are shown in Table 4 and Figure 2.

### 2.6. Three-Point Bending Test

We detected no influence of stiripentol administration on bone mechanical properties in rat femur, but observed decreased values of Young’s modulus and bending stiffness in rat tibia in the STP-receiving group in comparison to the control group (Table 5).

## 3. Discussion

The available data suggest that not only older antiepileptic drugs but also the newer AEDs may influence bone tissue [1,24,25]. For instance, topiramate given to rats at a daily dose of 20 mg/kg for 12 weeks decreased bone formation [26], whereas gabapentin at a daily dose of 150 mg/kg decreased bone formation, increased bone resorption and induced the deterioration of cancellous bone structure [27]. Takahashi et al. [28] noticed bone loss as a result of accelerated bone resorption in rats after the 5 week administration of zonisamide at a dose of 80 mg/kg/day. Nowińska et al. [29] studied the impact on bones of a daily 250 mg/kg dose of vigabatrin given for 4 weeks and reported that the growth of cortical bone was inhibited and the cancellous bone histomorphometric parameters were worsened.

**Figure 3:**
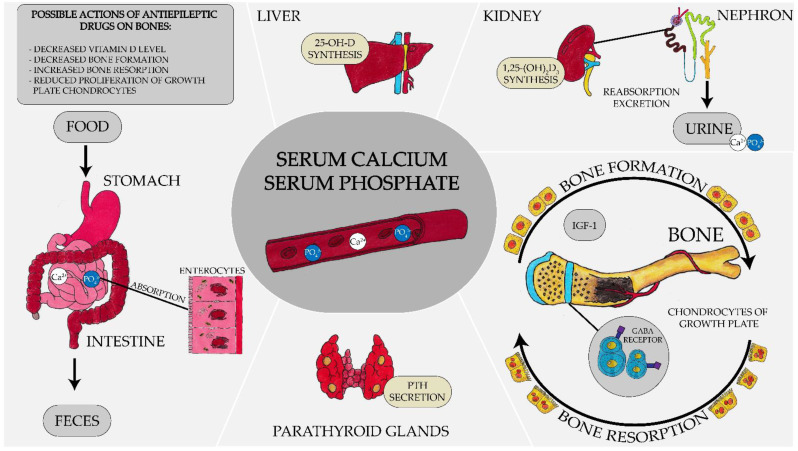
Mechanisms regulating serum calcium and phosphate levels. Serum calcium (Ca^2+^) and phosphates (PO_4_^3−^) levels depend on intestinal absorption, kidney excretion and release/deposition in bones. Parathormone (PTH) and 1,25-dihydroxyvitamin D_3_ are the most important factors regulating Ca^2+^ and PO_4_^3−^ serum levels. Vitamin D increases intestinal Ca^2+^ and PO_4_^3−^ absorption. PTH acts similarly, but its action is indirect by increasing 1,25-dihydroxyvitamin D_3_ production. Both 25-hydroxyvitamin D and 1,25-dihydroxyvitamin D_3_ increase Ca^2+^ and PO_4_^3−^ reabsorption in the kidneys, whereas PTH increases reabsorption of calcium but increases kidney excretion of phosphates. In bones, the effect of PTH depends on the dose/concentration. In high doses, it causes Ca^2+^ and PO_4_^3−^ reabsorption from bones and low doses it may increase the formation of bones. Similarly, the dual action of 1,25-dihydroxyvitamin D_3_ on Ca^2+^ and PO_4_^3−^ reabsorption has been described. Vitamin D acts on the parathyroid gland and regulates PTH secretion. Summing up, the net effect of PTH action is increased calcium with decreased phosphates in the serum and the net effect of vitamin D is increased calcium and phosphates in serum.

This study examines the impact of the long-term administration of stiripentol (STP) on bone in rats. Among laboratory parameters analyzed, we observed a significantly higher serum level of inorganic phosphate and a reduced level of 1,25-dihydroxyvitamin D_3_ in rats receiving STP for 24 weeks in comparison to the control group. Serum inorganic phosphate level is maintained by a variety of factors, including hormones [30,31] (Figure 3). In physiology, dietary phosphorus intake is counterbalanced by excretion. Stiripentol (Diacomit) did not contain any additional phosphorus. Rats in the control group and in the STP-administered group were fed the same balanced animal feed. The kidneys are involved in maintaining phosphates balance as they are responsible for phosphates elimination [32].

25-hydroxyvitamin D is synthesized in the liver from its precursor by enzymes which have the activity of 25-hydrolases. Later 25-OH-D is metabolized to 1,25-dihydroxyvitamin D_3_ in kidneys [33]. In our study we did not find any significant differences in 25-OH-D levels between the STP group and the control group in the 8th, 16th and 24th week of the experiment. Proximal tubules are the place of active vitamin D synthesis via 1α-hydroxylase, which is why tubular atrophy may contribute to 1,25-dihydroxyvitamin D_3_ deficiency [33]. However, there are other reasons that may result in a decreased level of 1,25-dihydroxyvitamin D_3_ in serum, e.g., the inhibitory effect on the activity of synthesizing enzymes and accelerated catabolism of 1,25-(OH)_2_D_3_ [34].

Many drugs used in clinical practice may cause various kidney injuries [35,36,37,38,39]. Both the structure of the kidneys (vascular density and blood flow are much higher than in many other tissues) and kidney function (excretion of metabolic wastes) make this organ very sensitive to endotoxins and exotoxins [40]. Drugs may injure the kidneys, resulting in glomerulopathy and/or tubular dysfunction such as tubulopathy or interstitial nephritis, with the proximal tubules epithelium being especially susceptible. Stiripentol is considered a relatively safe agent in animal toxicological studies, with a median lethal dose (LD_50_) after oral administration for rats of about 3 g/kg [10]. So far, it has been reported that repeated exposure to stiripentol at daily doses higher than 200 mg/kg is associated with acute tubular necrosis in the kidneys [10]. It cannot be excluded that in the development of renal pathology the cumulative dose may be important, even if daily doses are not particularly high. In our study, we did not observe any increase in the serum creatinine level. However, elevation of creatinine is a late marker of kidney dysfunction [41]. Creatinine is mainly excreted by glomerular filtration and less by tubular secretion [42], therefore it is not an insensitive marker of tubular injury [43]. An increase in the serum creatinine level is found only after damage to at least 50% of the nephron mass [43]. Currently, many studies focus on the role of neutrophil gelatinase-associated lipocalin (NGAL), a small glycoprotein expressed by neutrophils, renal tubular cells or podocytes as a marker of tubular damage [44,45]. Tubulointerstitial injury results in NGAL expression in tubular epithelial cells, which may reflect the level of kidney dysfunction. It was found that renal injury may be predicted based on urinary NGAL detection before changes in GFR are detectable [40,46]. Less is known about plasma NGAL determination and its predictive capability, but some studies suggest the inverse correlation of plasma NGAL level and GFR in children with kidney disease or in the model of diabetic nephropathy or glomerulonephritis [45,46,47]. Both, urinary and plasma NGAL levels are good predictors of kidney injury and progression of chronic kidney disease [46,48]. In our experiment, after 24 weeks of stiripentol administration at a dose of 200 mg/kg/day, we found significantly higher serum levels of NGAL compared to the control group, which may suggest kidney injury despite no significantly different creatinine levels existing in the control and stiripentol group. Our results suggest the need for further detailed studies to evaluate the long-term impact of stiripentol on kidneys function and/or structure.

Long-standing deficiency of vitamin D decreases the bone mineral density [49,50]. However, in the reported study, we did not detect decreased BMD in the stiripentol-receiving animals. It cannot be ruled out that longer observations are needed to evaluate the influence of stiripentol on BMD. This study demonstrated a lower trabecular thickness of the tibia in the stiripentol-receiving group, as evidenced by micro X-ray computed tomography. The consequence of this is a reduction in the bone volume fraction. Additionally, we observed a change in a trabeculae character from laminar to rod-like in the tibia (a higher structure model index in the STP group) and a higher trabecular pattern factor. These changes suggest the unfavorable effect of stiripentol on the microarchitecture of the trabecular bone. We did not observe any changes in the mCT scans of the femur nor in the third lumbar vertebra. In rats osteoporotic changes are found in the tibia much earlier than in other bones [23]. This may explain the fact that we found significant changes in bone microarchitecture only in tibias.

Sex hormones play a crucial role in bone homeostasis in males. Androgens stimulate the proliferation, differentiation and maturation of osteoblasts; inhibit osteoclasts recruitment; and influence the bidirectional interaction between osteoblasts and osteoclasts [51,52]. A decreased sex hormones level exacerbates bone mass loss and promotes osteoporosis [52]. In epileptic patients treated with AEDs, disorders of endocrine glands function and impaired fertility were described [53,54] The etiology of these phenomena is multifactorial. The impact of the disease (epilepsy), psychological stress and the use of antiepileptic drugs should be mentioned as very important, among other factors [55,56]. During epileptic seizures, the release of hypothalamus and pituitary gland hormones changes. Pulsatile secretion of hormones may be decreased or increased depending on which region of the brain is affected by the epileptic attack [57]. Psychosocial stress in epilepsy may play an important role in hypogonadism. It is well documented that chronic stress increases ACTH release, which, in turn, inhibits gonadotropins release and reproductive functions [55]. Antiepileptic drugs can disturb the levels of many various hormones. Through their action in the central nervous system, AEDs can modify neuromediators synaptic level and neuronal excitability and, in this fashion, AEDs may alter the hypothalamus-pituitary gland-target organs axis. Additionally, some AEDs increase sex-hormone binding globulin (SHBG) synthesis in the liver. As a consequence, bioactive testosterone and/or estrogens levels may be decreased [53].

Eschbach et al. demonstrated a reduced weight and height growth trend in a cohort of children suffering from Dravet syndrome. Endocrine dysfunction in the form of low levels of IGF-1 and testosterone were detected in a subset of the group studied [20]. In our current study, body weight, tibial and femoral length and diameter as well as the IGF-1 level in rats were not significantly different between the STP-receiving group and the corresponding control group. In our previously published paper, we did not find any decrease in the serum testosterone level nor any increase in the serum SHBG level in epilepsy-free rats receiving stiripentol. Furthermore, in the 4th week of the study, the serum testosterone level and testosterone/SHBG ratio were significantly higher in the STP group in comparison to the control group [58]. It may be suggested that the decrease in IGF-1 and testosterone levels in patients with Dravet syndrome, observed in a cohort study performed by Eschbach et al. [20], may not be related to stiripentol administration.

The changes in tibial bone microarchitecture in rats receiving stiripentol observed in our study may result from a decreased active vitamin D level and hyperphosphatemia [59], but may also be the consequence of the direct impact of STP on bones. STP revealed the pharmacological action by allosteric changes of the GABA_A_ receptors [9] and modulates GABA-ergic transmission [11]. GABA_A_ and GABA_B_ receptors have been identified in chondrocytes in the growth plate [60]. In vitro studies indicate GABA is a factor regulating in the autocrine/paracrine way and the state of the growth plate, which is the site of longitudinal bone growth [61]. Tamayama et al. [61,62] reported that in the chondrocytes of the growth plate in rat tibia, both GABA and GABA synthetizing enzymes (glutamate decarboxylases) are present. Therefore, it can be hypothesized that the influence of stiripentol on bone microarchitecture can be associated with its influence on GABA transmission.

Whole-bone mechanical behavior depends on bone mass, size, geometry and the structure of bone [63]. In our study, Young’s modulus, flexural strength and the stiffness of rat femur in a three-point bending test were not significantly different in the control and STP group. It is therefore a consequence of the lack of differences in macrometric femur measurements, bone mineral density of femur and femur microarchitecture between the groups studied. However, in tibia, lower values of Young’s modulus and lower bending stiffness were found in the three-point bending test which suggests worsening of the mechanical properties of tibia and these are consistent with rearrangement of tibial microarchitecture found micro-computed tomography.

## 4. Materials and Methods

### 4.1. Ethical Statements and Animals

The study was approved by the Local Ethics Committee for Animal Experiments at Hirszfeld Institute of Immunology and Experimental Therapy of the Polish Academy of Science in Wroclaw (Approval code 59/2018, 20 June 2018). All actions and procedures in the study were consistent with ethical standards.

The rats were bred and kept in the Animal Laboratory Facility at Wroclaw Medical University where, after required acclimatization period, the main study was conducted. During the experiment, the animals were housed two per cage, on a 12 h/12 h light-dark cycle, at a constant ambient temperature (22 °C), with unlimited access to drinking water and standard feed for laboratory rats.

### 4.2. Design of the Study

Twenty-four 7 week old, male Wistar rats were randomly divided into two experimental groups (12 animals in each group): group C (control group) receiving 0.9% sodium chloride 4 mL/kg/day (0.9% Sodium Chloride-Braun, Braun, Germany); and STP group receiving stiripentol 200 mg/kg/day [64,65,66] (Diacomit, Biocodex, France) dissolved in 0.9% Sodium Chloride-Braun 4 mL/kg/day. Stiripentol and/or 0.9% sodium chloride were administered once daily for 24 consecutive weeks.

The dose of stiripentol in rats was calculated according to the guide for dose conversion between animals and human [67]. The dose of STP for human of 30–50 mg/kg/day correspond to doses of 158–264 mg/kg/day in rats. The stiripentol dose of 200 mg/kg/day in rats chosen in our study is equal to 38 mg/kg/day in humans. The 24 week usage of the drug was selected to assess the possible adverse effects of long-term administration which is consistent with the available literature [68,69,70]. The sample size (number of rats in each group) was estimated before the experiment and is based on bone mineral density (BMD) values. With an assumed population average of BMD of 0.27 ± 0.008 g/cm^2^, an assumed minimal difference of 0.01 g/cm^2^ with the maximum probability of making the first type error of α = 0.05 and an assumed target power of the test of β = 0.8, the required group size (n) is then 12.

After 8 weeks and 16 weeks of the administration of stiripentol and/or 0.9% sodium chloride, blood samples from the tail vein were collected.

After 24 weeks of administering stiripentol and/or 0.9% sodium chloride, under general anesthesia (with intraperitoneal ketamine 60 mg/kg and xylazine 10 mg/kg), blood samples were taken via cardiac puncture and immediately thereafter (without waking the animals from anesthesia) the rats were euthanized by cervical dislocation (C6–C7). Next, the right tibia and right femur were collected and adjacent tissues were removed. The lumbar spine with ligaments was harvested.

The right tibia and right femur were weighed and measured. Densitometry and micro-computed tomography (mCT) of the right tibia and right femur were performed. Then a three-point bending test of the right tibia and the right femur was conducted. The bone mineral density of the lumbar spine at the level of L1–L4 with adjacent ligaments was evaluated densitometrically. Subsequently, the third lumbar vertebra (L3) was isolated from the surrounding tissues and a mCT was performed.

### 4.3. Serum Parameters

Blood samples were centrifuged for 10 min at 4000× *g* in 4 °C using laboratory centrifuge MPW-350R (MPW Med. Instruments, Warsaw, Poland). The serum samples obtained were frozen and stored in −80 °C for further laboratory assessments.

In serum, levels of N-terminal Propeptide of type I Procollagen (PINP), osteoclast-derived Tartrate-Resistant Acid Phosphatase form 5b (TRAP), 25-hydroxyvitamin D (25-OH-D), 1,25-dihydroxyvitamin D_3_ (1,25-(OH)_2_D_3_), parathormone (PTH), insulin-like growth factor 1 (IGF-1) and neutrophil gelatinase-associated lipocalin (NGAL) were determined using ELISA kits, following their respective manufacturers’ instructions (Rat/Mouse PINP EIA AC-33F, Immunodiagnostic Systems Limited, Boldon, UK; Rat TRAP^TM^ ELISA SB-TR102, Immunodiagnostic Systems Limited, Boldon, UK; Rat 25-hydroxyvitamin D ELISA E1445Ra, Bioassay Technology Laboratory, Shanghai, China; Rat 1,25-dihydroxyvitamin D_3_ ELISA E0000Ra, Bioassay Technology Laboratory, Shanghai, China; Parathyroid hormone ELISA CEA866Ra, Cloud-Clone Corp., TX, USA; Insulin Like Growth Factor 1 ELISA REF SEA050Ra Cloud-Clone Corp., TX, USA; Rat NGAL ELISA Kit ELK5638, ELK Biotechnology, Wuhan, China).

Concentrations of total calcium, inorganic phosphate, creatinine and activities of hepatic transaminases were assessed in a certified commercial laboratory using an Architect plus ci4100 device from Abbott and the following tests: Calcium Architect/Aeroset REF 3L79-21 and 3L79-31 304328/R1; Phosphorus Architect REF 7D71 305532/R02; Creatinine (enzymatic) Architect REF 8L24-31 and 8L24-41 G6-6497/R06 B8L24P; Aspartate aminotransferase Architect REF 7D81-22; Alanine aminotransferase Architect REF 7D-56-21; Abbott, IL, USA).

### 4.4. Macrometric Measurements

Directly after collection, the right-side tibias and femurs were carefully cleared of adjacent tissues and weighed on AS 60/220/C/2 (Radwag, Radom, Poland) electronic scales. Tibial index and femoral index values were calculated using the following formulas

Tibial index = weight of right tibia (g)/weight of rat on the last day of study (g)

Femoral index = weight of right femur (g)/weight of rat on the last day of study (g)

Size measurements of bones were conducted using Pro electronic calipers (Pro sp. z o.o., Bielsko-Biała, Poland) with a 0.01 mm resolution.

### 4.5. Densitometry

Bone densitometry was performed using Hologic Discovery W 81,507 (Hologic Inc., Marlborough, MA, USA) equipment with software for small animals. Bone mineral density (BMD) was expressed in grams per square centimeter (g/cm^2^). Tibial BMD, femoral BMD and L1–L4 BMD were assessed. Before measurements were made, the densitometer was calibrated with proper phantoms.

### 4.6. Micro X-ray Computed Tomography

Structural properties were examined using SkyScan 1172 (Bruker, Kontich, Belgium) computed microtomography. Each sample of long bone (tibia and femur) was registered with a resolution of 9 μm at a lamp value of 74 kV/133 μA, using a 0.5 mm Al filter and a rotation angle of 0.45°. Vertebra body was registered using a RTG lamp with properties of 80 kV/124 μA.

**Figure 4:**
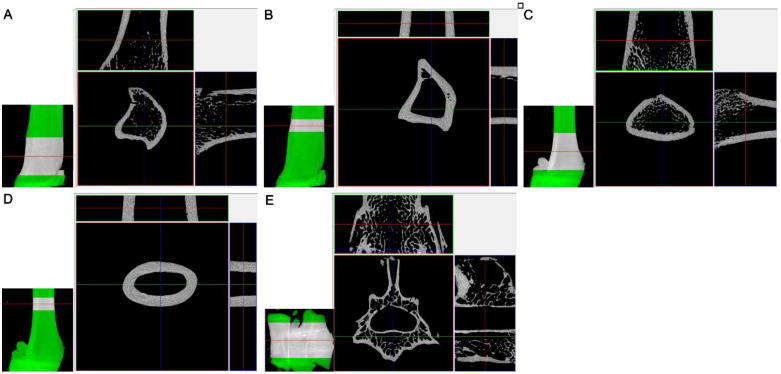
Analyzed regions of interest (ROI) of the tibia (proximal metaphysis), the femur (distal metaphysis) and the third lumbar vertebra. (**A**) ROI of the cancellous bone of tibia; (**B**) ROI of the cortical bone of tibia; (**C**) ROI of the cancellous bone of femur; (**D**) ROI of the cortical bone of femur; (**E**) ROI of the cancellous tissue of vertebra.

The region of interest (ROI) (Figure 4) was selected in accordance with the guidelines for assessment of bone microstructure in rodents [71] in the CTAn program (Bruker^®^), with method note MCT-003 with correction of the long axis of the bone and determination of the growth plate area [71]. For the long bone, two regions were selected: the cancellous bone and the cortical bone. The regions were defined as 400 and 200 intersections from the location of the growth plate for the cancellous bone and cortical bone, respectively. For a resolution of 9 µm, this is equivalent to a length of approximately 3.6 mm and 1.8 mm, respectively. For the cancellous tissue of vertebra body, the regions had a length of 300 intersections, which is equivalent to approximately 2.7 mm.

Quantitative analysis of the cancellous bone structure involved determining the bone volume fraction (BV/TV), bone surface density (BS/TV), trabecular thickness (Tb.Th), trabecular number (Tb.N), trabecular separation (Tb.Sp), trabecular pattern factor (Tb.Pf), structure model index (SMI), degree of anisotropy (DA), connectivity density (Conn.D) and total porosity (Po.tot). For the cortical bone, the analysis took into account the average cortical thickness (Ct.Th), the total cross-sectional area inside the periosteal envelope (Tt.Ar), cortical bone area (Ct.Ar) and the cortical area fraction (Ct.Ar/Tt.Ar) [71].

### 4.7. Three-Point Bending Test

The investigations of the mechanical properties were carried out in a three-point bending test using a MTS 858 MiniBionix machine (Eden Prairie, MN, USA). For mechanical tests of tibia, the spacing of the bottom prism was equal to 20 mm (l_0_) and the load was applied to the tibia in 58% of the bone length from distal epiphysis on the lateral surface. The load speed during bending test was 6 mm/min. For the femur, the spacing of the bottom prisms was equal to 18 mm (l_0_) and the load was applied to the mid part of the femoral bone on the posterior surface from the front of the bone. The load speed during bending was 1 mm/min. Mechanical tests conducted in the three-point bending test allowed mechanical parameters to be determined: Young’s modulus, bending strength and bending stiffness. The values of the parameters were determined using classical formulas [72]. In order to calculate Young’s modulus, the cross-section areas of the tibia and the femur sample were required to be measured. The cross-section area of each sample was determined at the point where the sample broke. The cross-section was approximated with the area of the smallest ellipse that outlines the sample. The area was calculated from the measurements of the length of ellipse’s axis using a Zeiss Stereo Discovery V20 stereo microscope (Jena, Germany).

### 4.8. Statistical Analysis

Statistical analysis was performed using STATISTICA software, version 13.3 (Dell Software Inc., Round Rock, TX, USA). The level of significance was set at *p* < 0.05. The results are presented as follows: median (lower quartile—upper quartile), because the data were not normally distributed. All statistical analyses were performed by means of non-parametric tests.

## 5. Conclusions

Our study showed that rats given stiripentol for 24 weeks had significantly impaired tibial bone microarchitecture and biomechanical properties, decreased 1,25-dihydroxyvitamin D_3_, increased inorganic phosphate and increased neutrophil gelatinase-associated lipocalin level in serum. Stiripentol may be used as a support therapy in Dravet syndrome in children and adults. Considering our findings, further prospective human studies seem to be justified and neurologists should be encouraged to monitor the vitamin D level and calcium-phosphate homeostasis as well as kidneys function in patients taking stiripentol.

## Figures and Tables

**Table 1:** The effect of administration of stiripentol on serum parameters (PINP-N-terminal propeptide of type I procollagen; TRAP—tartrate-resistant acid phosphatase form 5b; 25-OH-D-25-hydroxyvitamin D; NGAL—neutrophil gelatinase-associated lipocalin; STP group—group receiving stiripentol). Results presented as: median (lower quartile—upper quartile).

	Serum Parameters	Control Group	STP Group	*p* Value
**Week 8**	PINP (ng/mL)	3.17 (2.37–3.54)	2.94 (2.04–3.37)	*0.005*
	TRAP (U/L)	0.92 (0.68–0.94)	0.74 (0.66–0.91)	NS
	25-OH-D (nmol/L)	5.51 (5.12–5.76)	6.17 (3.84–6.71)	NS
**Week 16**	PINP (ng/mL) *	2.61 (1.76–2.73)	1.70 (1.40–1.87)	NS
	TRAP (U/L)	0.77 (0.63–0.82)	0.78 (0.69–0.89)	NS
	25-OH-D (nmol/L)	6.12 (5.08–6.59)	5.45 (4.41–5.91)	NS
	Total calcium (mg/dL)	8.00 (7.40–9.10)	7.40 (7.00–8.90)	NS
	Inorganic phosphate (mg/dL)	3.80 (3.60–4.80)	4.40 (3.70–5.20)	NS
**Week 24**	PINP (ng/mL)	0.84 (0.78–1.00)	0.78 (0.69–0.99)	NS
	TRAP (U/L)	0.69 (0.64–0.84)	0.82 (0.66–0.90)	NS
	25-OH-D (nmol/L)	5.66 (5.18–5.97)	5.25 (4.47–6.44)	NS
	1,25-dihydroxyvitamin D_3_ (nmol/L) *	1.42 (1.37–1.69)	1.33 (1.25–1.46)	*0.04*
	Parathormone (pg/mL)	536.1 (417.0–754.2)	464.7 (340.4–570.0)	NS
	IGF-1 (ng/mL)	1.58 (1.29–2.38)	1.35 (1.18–1.47)	NS
	Total calcium (mg/dL)	9.65 (9.35–9.95)	9.70 (9.50–10.05)	NS
	Inorganic phosphate (mg/dL) *	4.40 (3.85–5.15)	5.80 (4.95–6.45)	*0.015*
	Creatinine (mg/dL)	0.29 (0.27–0.31)	0.30 (0.28–0.32)	NS
	NGAL (ng/mL) *	0.27 (0.26–0.29)	0.30 (0.27–0.31)	*0.03*
	Aspartate aminotransferase (U/L)	189 (139.5–292)	198 (139.5–263.5)	NS
	Alanine aminotransferase (U/L)	58 (48–70.5)	49 (39.5–63.5)	NS

* *p* < 0.05 indicates significant difference; NS—not significant.

**Table 2:** The effect of 24 weeks administration of stiripentol on macrometric parameters of bones (STP group—group received stiripentol for 24 weeks). Results presented as: median (lower quartile—upper quartile); NS—not significant.

Bone Macrometric Parameters	Control Group	STP Group	*p* Value
Tibial index (-)	0.0032 (0.0029–0.0033)	0.0032 (0.0029–0.0033)	NS
Tibial weight (g)	0.964 (0.930–1.044)	0.9210 (0.895–0.960)	NS
Tibia length (mm)	43.33 (42.62–43.77)	42.57 (42.50–43.05)	NS
Mid-tibial diameter (mm)	3.735 (3.625–3.850)	3.720 (3.640–3.945)	NS
Femur index (-)	0.0021 (0.0021–0.0023)	0.0021 (0.0020–0.0023)	NS
Femur weight (g)	1.372 (1.334–1.431)	1.322 (1.270–1.406)	NS
Femur length (mm)	39.77 (39.38–40.37)	39.70 (39.21–40.13)	NS
Mid-femoral diameter (mm)	4.815 (4.710–4.915)	4.680 (4.640–4.970)	NS

**Table 3:** The effect of 24 weeks administration of stiripentol on bone mineral density (BMD) (group STP—group received stiripentol for 24 weeks). Results presented as: median (lower quartile—upper quartile); NS—not significant.

Bone Mineral Density	Control Group	STP Group	*p* Value
Tibial BMD (g/cm^2^)	0.240 (0.231–0.243)	0.234 (0.232–0.241)	NS
Femoral BMD (g/cm^2^)	0.291 (0.279–0.294)	0.282 (0.275–0.290)	NS
L1–L4 BMD (g/cm^2^)	0.340 (0.334–0.360)	0.324 (0.320–0.342)	NS

**Table 4:** The effect of 24 weeks administration of stiripentol on parameters of bone assessed using micro X-ray computed tomography (BV/TV—bone volume fraction; BS/TV—bone surface density; Tb.Th—trabecular thickness; Tb.N—trabecular number; Tb.Sp—trabecular separation; Tb.Pf—trabecular pattern factor; SMI—structure model index; DA—degree of anisotropy; Conn.D—connectivity density; Po.tot—total porosity; Ct.Th—average cortical thickness; Tt.Ar—total cross-sectional area inside the periosteal envelope; Ct.Ar—cortical bone area; Ct.Ar/Tt.Ar—cortical area fraction; L3 vertebra—third lumbar vertebra; STP group—group received stiripentol for 24 weeks). Results presented as: median (lower quartile—upper quartile).

		Bone Parameters	Control Group	STP Group	*p* Value
**Tibia**	Cancellous bone	BV/TV (%) *	9.28 (8.63–10.20)	8.02 (7.70–8.60)	*0.03*
		BS/TV (mm^3^/mm^2^)	4.62 (4.25–5.54)	4.37 (4.17–5.42)	NS
		Tb.Th (mm) *	0.078 (0.07–0.08)	0.075 (0.07–0.08)	*0.03*
		Tb.N (1/mm)	1.20 (1.13–1.39)	1.08 (1.03–1.47)	NS
		Tb.Sp (mm)	0.70 (0.63–1.10)	0.64 (0.46–0.80)	NS
		Tb.Pf (1/mm) *	19.76 (18.13–20.27)	21.26 (20.21–23.37)	*0.02*
		SMI (-) *	2.30 (2.23–2.32)	2.40 (2.32–2.59)	*0.006*
		DA (-)	1.47 (1.44–1.62)	1.55 (1.32–1.85)	NS
		Conn.D (1/mm^3^)	33.16 (26.38–37.31)	23.67 (17.84–37.33)	NS
		Po.tot (%)	90.72 (89.81–91.37)	91.75 (89.74–92.47)	NS
	Cortical bone	Cr.Th (mm)	0.65 (0.63–0.67)	0.65 (0.62–0.66)	NS
		Tt.Ar (mm^2^)	52.49 (50.37–53.79)	51.28 (50.42–54.72)	NS
		Ct.Ar (mm^2^)	65.79 (63.57–67.04)	64.55 (61.95–67.72)	NS
		Tt.Ar/Ct.Ar (%)	80.40 (78.92–81.21)	79.56 (78.59–81.77)	NS
**Femur**	Cancellous bone	BV/TV (%)	14.47 (12.95–16.76)	12.63 (11.58–18.95)	NS
		BS/TV (mm^3^/mm^2^)	6.55 (6.14–7.93)	6.04 (5.65–8.56)	NS
		Tb.Th (mm)	0.079 (0.77–0.085)	0.077 (0.076–0.081)	NS
		Tb.N (1/mm)	1.77 (1.67–2.11)	1.61 (1.51–2.33)	NS
		Tb.Sp (mm)	0.60 (0.49–0.86)	0.56 (0.45–0.78)	NS
		Tb.Pf (1/mm)	15.35 (13.94 - 18.91)	16.64 (15.46–18.59)	NS
		SMI (-)	1.92 (1.82–2.11)	2.03 (1.93–2.19)	NS
		DA (-)	1.25 (1.19–1.38)	1.32 (1.26–1.44)	NS
		Conn.D (1/mm^3^)	65.20 (59.08–84.63)	54.02 (51.29–86.07)	NS
		Po.tot (%)	85.53 (85.19–89.39)	87.37 (85.27–90.20)	NS
	Cortical bone	Cr.Th (mm)	0.75 (0.74–0.78)	0.76 (0.72–0.80)	NS
		Tt.Ar (mm^2^)	52.77 (52.03–54.57)	50.97 (49.92–54.05)	NS
		Ct.Ar (mm^2^)	62.00 (60.41–63.56)	60.41 (58.29–63.57)	NS
		Tt.Ar/Ct.Ar (%)	86.23 (85,58–86.76)	85.55 (84.74–86.38)	NS
**L3 vertebra**	Cancellous bone	BV/TV (%)	19.08 (17.59–21.82)	17.79 (16.57–22.69)	NS
		BS/TV (mm^3^/mm^2^)	8.67 (7.88–9.69)	8.42 (7.67–10.73)	NS
		Tb.Th (mm)	0.078 (0.077–0.079)	0.076 (0.075–0.081)	NS
		Tb.N (1/mm)	2.44 (2.22–2.82)	2.36 (2.15–3.03)	NS
		Tb.Sp (mm)	0.27 (0.26–0.33)	0.27 (0.25–0.37)	NS
		Tb.Pf (1/mm)	12.42 (11.95–15.39)	13.47 (12.88–15.86)	NS
		SMI (-)	1.65 (1.67–1.95)	1.73 (1.70–1.97)	NS
		DA (-)	1.44 (1.35–1.61)	1.41 (1.34–1.52)	NS
		Conn.D (1/mm^3^)	74.70 (63.79–108.88)	70.87 (61.4–118.15)	NS
		Po.tot (%)	80.92 (80.21–86.03)	82.21 (80.13–86.07)	NS

* *p* < 0.05 indicates significant difference; NS—not significant.

**Table 5:** The effect of 24 weeks administration of stiripentol on mechanical properties of rat tibia and femur in three-point bending test (STP group—group received stiripentol for 24 weeks). Results presented as: median (lower quartile—upper quartile); NS—not significant.

	Bone Mechanical Properties	Control Group	STP Group	*p* Value
**Tibia**	Young’s modulus (GPa)	11.12 (10.02–12.67)	9.51 (8.67–11.24)	*0.044*
	Bending strength (MPa)	251.66 (237.96–273.53)	244.33 (232.60–248.92)	NS
	Bending stiffness (Nm^2^)	0.0431 (0.0401–0.0531)	0.0393 (0.0333–0.0404)	*0.024*
**Femur**	Young’s modulus (GPa)	6.13 (5.67–8.78)	6.31 (5.72–6.89)	NS
	Bending strength (MPa)	166.76 (157.44–184.17)	162.07 (158.06–183.15)	NS
	Bending stiffness (Nm^2^)	0.06 (0.05–0.08)	0.05 (0.05–0.07)	NS

## Data Availability

The data underlying this article will be shared upon request to the corresponding author.
